# Assessment of addictive behavior in patients with schizophrenia

**DOI:** 10.1192/j.eurpsy.2023.2288

**Published:** 2023-07-19

**Authors:** M. Zbidi, W. Bouali, H. Baba, M. Kacem, S. Younes, L. Zarrouk

**Affiliations:** Psychiatry department, Taher sfar University hospital of mahdia tunisia, Mahdia, Tunisia

## Abstract

**Introduction:**

Schizophrenia, a chronic and complex psychiatric pathology, can be isolated. However, it may have other comorbidities and thus be accompanied by addictive behaviors complicating their management.

**Objectives:**

to estimate the prevalence and identify the characteristics of addictive behavior among patients with schizophrenia.

**Methods:**

A retrospective study of 151 patients with schizophrenia and hospitalized in the psychiatry department of the Taher Sfar university hospital in Mahdia from January 2017 to December 2021.

**Results:**

The mean age of the patients was 39.8 ± 11.23 years with a predominance of age group 36-45 years (38.4%). All of the patients were males . Three quarters of patients (75.5%) were users of psychoactive substances (PSA): nearly three quarters (72.8%) dependent on tobacco, more than a third (39.7%) dependent on alcohol, more a quarter (29.1%) dependent on cannabis and almost a quarter (26.5%) dependent on other SPA. In more than half of the cases (54.4%), the age at which SPA consumption began was between 16 and 25. SPA use preceded the onset of schizophrenia in 62.3% of case. The relationship with the entourage was marked by hetero-aggressiveness in 77.5% of the patients, a withdrawal from the entourage for 16.6% of the patients and a conflict for 5.3% of the patients. The impact on the relationship with oneself was marked by self-aggressiveness in 18.5% of patients. Regarding professional impact, three quarters of patients (76.1%) had to stop working. The majority of patients (84.1%) continued their usual treatment, while 15.2% of patients stopped it. In only one patient increased doses were necessary.

**Conclusions:**

Subjects suffering from schizophrenia are particularly vulnerable to addictions, mainly to tobacco and alcohol. They are therefore a group at greater risk of harmful effects of psychoactive substances and at worsening the clinical course of their psychiatric illness. Screening and treatment measures their addictive behaviors early on, even before schizophrenia sets in, should be offered.

**Disclosure of Interest:**

None Declared

